# Supplementary material for: Identifying where Japanese agriculture is most at risk: A longitudinal analytical framework based on municipal boundaries as of 1950 for workforce decline and aging (2005–2020)
Source: PLoS One. 2026 Apr 1;21(4):e0334403. doi: 10.1371/journal.pone.0334403 (PMC13042669; doi:10.1371/journal.pone.0334403)
Supplement: S1 File — This file contains: S1 Appendix, land-type definitions and classification procedures; S2 Appendix, additional information on the methods used; S1 Table, estimated marginal means (response scale) by land-type combination for population decrease and elderly proportion; and S2 Table, Tukey-adjusted pairwise contrasts among the 12 land-type combinations (link scale). (DOCX) [file pone.0334403.s001.docx]

## **S1 Appendix. Land-type definitions and classification procedures**

We used the MAFF Agricultural Area Classification. A statistical zoning scheme defined for each municipality and sub-municipality based on land-use characteristics for agricultural statistics and policy planning. The classification consists of a primary category (landtype1: Urban-Flatland-Intermediate-Mountainous Agricultural Area) and a secondary category (landtype2: Paddy-Mixed-Upland Dominated Type) determined by stable land-use indicators and the paddy-field ratio.

### **Primary classification (landtype1)**

Urban Area

- If DID area share within habitable land ≥ 5% and, (population density ≥ 500 persons/km² or DID population ≥ 20,000), then Urban.
- Or if residential land share within habitable land ≥ 60% and population density ≥ 500 persons/km², then Urban, exclude units with forest cover ≥ 80%.

Flatland Agricultural Area

- If cropland ratio ≥ 20% and forest cover < 50%, then Flatland, exclude units where the share of steep-slope paddy (≥1/20) + steep-slope upland (≥8°) within total cropland is ≥ 90%.
- Or if cropland ratio ≥ 20% and forest cover ≥ 50%, then Flatland, when the same “steep-slope cropland share” is < 10%.

Intermediate Agricultural Area

- If cropland ratio < 20% and not classified as Urban or Mountainous, then Intermediate.
- Or if cropland ratio ≥ 20% and not classified as Urban or Flatland, then Intermediate.

Mountainous Agricultural Area

- If forest cover ≥ 80% or, (forest cover ≥ 80% AND cropland ratio < 10%), then Mountainous.

### **Secondary classification (landtype2)**

Paddy type: paddy-field ratio ≥ 70%

Mixed (paddy–upland) type: 30%–70%

Upland type: < 30%

### **Decision order (classification sequence)**

Following the MAFF specification, the primary agricultural area category (landtype1) is assigned in a fixed sequence to ensure unique classification. In practice, areas are first screened for the Urban category, then for Mountainous, then for Flatland, and any remaining areas are assigned to Intermediate (Urban → Mountainous → Flatland → Intermediate).

### **Definition of DID (Densely Inhabited District)**

DID (Densely Inhabited District) is defined as an area formed by contiguous basic census units with a population density of at least 4,000 persons per km², and with a total (combined) population of 5,000 or more.

## **S2 Appendix. Additional information on the methods used**

### **Data and model**

We analyzed governmental agricultural statistics at the 1950 municipal boundary unit (sub-municipalities). Outcomes were the decline rate (2005–2020) and the proportion of the elderly (aged ≥75 years or above) in 2020 among core agricultural workers. For each outcome, we fitted a beta–logit GLMM:

$$beta \_ outcome \sim{landtype1}/{landtype2 + \left( 1 | region \_ block \right)}$$

Here, landtype1/landtype2 ≡ landtype1 + landtype1:landtype2; the main effect of landtype2 is not included. Reference levels were Lowland (landtype1) and Paddy-dominated (landtype2). Zero inflation was not used (zi=~0); dispersion was constant (disp=~1). Land-type definitions follow MAFF.

### **Estimated marginal means (EMMs, response scale)**

EMMs on the response scale for all 12 combinations of landtype1 × landtype2 are listed in S1 Table with 95% CIs and sample sizes (N). Figure 6 in the main manuscript visualizes the same EMMs as heatmaps. Pairwise differences among the 12 combinations were tested using Tukey-adjusted contrasts based on the EMMs. The results (link scale) are provided in S2 Table along with multiplicity-adjusted 95% CIs and *p*-values. Inference used asymptotic z-tests (df = ∞). Because the model uses landtype1/landtype2, the interpretation of landtype2 was within each landtype1 level; no landtype2 main effect is included.

### **Diagnostics**

We assessed model adequacy using DHARMa simulated residuals. For both outcomes, residual quantile–quantile patterns and residuals versus fitted values did not indicate deviations from the assumed beta–logit mean–variance structure. Simulation-based tests of residual uniformity and dispersion were non-significant. These checks supported the use of the beta-logit GLMM with a regional random intercept; inference therefore relied on asymptotic z methods (df = ∞).

### **Reproducibility**

All analyses were conducted in R using publicly available packages only: glmmTMB for fitting beta–logit GLMMs with a regional random intercept, emmeans for estimated marginal means and Tukey-adjusted pairwise contrasts, and DHARMa for residual diagnostics. No bespoke algorithms or user-defined estimation routines were employed. Given that the full model specification (formulae, reference levels, and nesting of landtype1/landtype2) is reported in the manuscript (Table 1 and Fig. 6), the results can be reproduced from the data with these packages alone.

### **S1 Table. Estimated marginal means (response scale) by land-type combination for population decrease and elderly proportion.**

| Outcome | Landtype1 | Landtype2 | N | Predicted mean (%) | Lower 95% Cl (%) | Upper 95% Cl (%) |
| --- | --- | --- | --- | --- | --- | --- |
| Decline | Urban | Paddy | 1121 | 40.6 | 37.7 | 43.5 |
|  |  | Mixed | 799 | 44.3 | 41.2 | 47.4 |
|  |  | Upland | 549 | 44.4 | 41.2 | 47.7 |
|  | Flatland | Paddy | 1741 | 38.3 | 35.6 | 41.1 |
|  |  | Mixed | 661 | 41.0 | 38.0 | 44.1 |
|  |  | Upland | 350 | 37.8 | 34.5 | 41.1 |
|  | Hilly | Paddy | 1631 | 41.4 | 38.5 | 44.3 |
|  |  | Mixed | 1039 | 45.9 | 42.9 | 48.9 |
|  |  | Upland | 536 | 47.2 | 44.0 | 50.5 |
|  | Mountainous | Paddy | 897 | 40.3 | 37.3 | 43.3 |
|  |  | Mixed | 557 | 48.3 | 45.0 | 51.5 |
|  |  | Upland | 291 | 54.1 | 50.5 | 57.7 |
| Elderly | Urban | Paddy | 1258 | 34.3 | 30.7 | 38.0 |
|  |  | Mixed | 850 | 33.4 | 29.8 | 37.1 |
|  |  | Upland | 592 | 31.1 | 27.7 | 34.8 |
|  | Flatland | Paddy | 1879 | 32.5 | 29.1 | 36.1 |
|  |  | Mixed | 666 | 32.4 | 28.9 | 36.2 |
|  |  | Upland | 355 | 28.2 | 24.9 | 31.9 |
|  | Hilly | Paddy | 1785 | 37.3 | 33.6 | 41.1 |
|  |  | Mixed | 1055 | 36.3 | 32.7 | 40.2 |
|  |  | Upland | 561 | 34.6 | 31.0 | 38.5 |
|  | Mountainous | Paddy | 978 | 38.9 | 35.2 | 42.9 |
|  |  | Mixed | 570 | 37.4 | 33.6 | 41.4 |
|  |  | Upland | 305 | 35.4 | 31.5 | 39.5 |

Predicted means (EMMs) from beta–logit GLMMs with a random intercept for region_block (14 groups). Values are on the response scale and reported as percentages where appropriate. “N” is the number of municipal units in each combination of topographical category (landtype1: Urban/Flatland/Hilly/Mountainous) and land-use type (landtype2: Paddy-dominated/Paddy-Upland-Mixed/Upland-dominated). 95% CIs are asymptotic. The reference combination is Lowland × Paddy. These numerical EMMs correspond to Fig. 6; omnibus tests are shown in Table 1 and pairwise contrasts in S2 Table.

### **S2 Table. Tukey-adjusted pairwise contrasts among the 12 land-type combinations (link scale).**

| Outcome | Contrast | Delta (logit) | Lower 95% CI (logit) | Upper 95% CI (logit) | z | Adjusted p |
| --- | --- | --- | --- | --- | --- | --- |
| Decline | PF – MxF | -0.114 | -0.240 | 0.013 | -2.937 | 0.128 |
|  | PF – UpF | 0.023 | -0.143 | 0.188 | 0.446 | 1 |
|  | PF – PU | -0.096 | -0.202 | 0.011 | -2.941 | 0.127 |
|  | PF – MxU | -0.248 | -0.367 | 0.129 | -6.793 | < 0.001 |
|  | PF – UpU | -0.253 | -0.396 | 0.110 | -5.764 | < 0.001 |
|  | PF – PH | -0.128 | -0.225 | 0.031 | -4.312 | 0.001 |
|  | PF – MxH | -0.311 | -0.420 | 0.202 | -9.304 | < 0.001 |
|  | PF – UpH | -0.367 | -0.505 | 0.228 | -8.667 | < 0.001 |
|  | PF – PM | -0.084 | -0.200 | 0.033 | -2.345 | 0.444 |
|  | PF – MxM | -0.408 | -0.543 | 0.273 | -9.873 | < 0.001 |
|  | PF – UpM | -0.641 | -0.817 | 0.465 | -11.926 | < 0.001 |
|  | MxF – UpF | 0.136 | -0.047 | 0.320 | 2.423 | 0.39 |
|  | MxF – UpU | 0.018 | -0.119 | 0.155 | 0.426 | 1 |
|  | MxF – MxU | -0.135 | -0.278 | 0.009 | -3.053 | 0.094 |
|  | MxF – UpU | -0.139 | -0.303 | 0.024 | -2.786 | 0.186 |
|  | MxF – PH | -0.014 | -0.145 | 0.117 | -0.354 | 1 |
|  | MxF – MxH | -0.197 | -0.335 | 0.060 | -4.695 | < 0.001 |
|  | MxF – UpH | -0.253 | -0.414 | 0.092 | -5.133 | < 0.001 |
|  | MxF – UpM | 0.030 | -0.116 | 0.176 | 0.675 | 1 |
|  | MxF – MxM | -0.294 | -0.453 | 0.135 | -6.052 | < 0.001 |
|  | MxF – UpM | -0.527 | -0.721 | 0.333 | -8.889 | < 0.001 |
|  | UpF – PU | -0.118 | -0.291 | 0.054 | -2.245 | 0.517 |
|  | UpF – MxU | -0.271 | -0.449 | 0.093 | -4.969 | < 0.001 |
|  | UpF – UpU | -0.276 | -0.468 | 0.083 | -4.68 | < 0.001 |
|  | UpF – UpH | -0.15 | -0.318 | 0.017 | -2.929 | 0.131 |
|  | UpF – MxH | -0.334 | -0.506 | 0.161 | -6.325 | < 0.001 |
|  | UpF – UpH | -0.389 | -0.578 | 0.200 | -6.736 | < 0.001 |
|  | UpF – UpM | -0.106 | -0.285 | 0.073 | -1.933 | 0.738 |
|  | UpF – MxM | -0.43 | -0.619 | 0.241 | -7.445 | < 0.001 |
|  | UpF – UpM | -0.664 | -0.881 | 0.446 | -9.983 | < 0.001 |
|  | PU – MxU | -0.152 | -0.280 | 0.024 | -3.889 | 0.006 |
|  | PU – UpU | -0.157 | -0.308 | 0.006 | -3.401 | 0.033 |
|  | PU – PH | -0.032 | -0.140 | 0.076 | -0.972 | 0.998 |
|  | PU – MxH | -0.215 | -0.335 | 0.095 | -5.862 | < 0.001 |
|  | PU – UpH | -0.271 | -0.417 | 0.125 | -6.071 | < 0.001 |
|  | PU – UpM | 0.012 | -0.113 | 0.137 | 0.32 | 1 |
|  | PU – MxM | -0.312 | -0.455 | 0.169 | -7.125 | < 0.001 |
|  | PU – UpM | -0.545 | -0.727 | 0.363 | -9.794 | < 0.001 |
|  | MxU – UpU | -0.005 | -0.161 | 0.151 | -0.101 | 1 |
|  | MxU – PH | 0.120 | -0.002 | 0.242 | 3.22 | 0.058 |
|  | MxU – MxH | -0.063 | -0.194 | 0.068 | -1.573 | 0.919 |
|  | MxU – UpH | -0.118 | -0.273 | 0.036 | -2.512 | 0.332 |
|  | MxU – UpM | 0.165 | 0.027 | 0.302 | 3.908 | 0.005 |
|  | MxU – MxM | -0.160 | -0.312 | 0.008 | -3.434 | 0.029 |
|  | MxU – UpM | -0.393 | -0.581 | 0.205 | -6.824 | < 0.001 |
|  | UpU – PH | 0.125 | -0.021 | 0.272 | 2.791 | 0.184 |
|  | UpU – MxH | -0.058 | -0.212 | 0.096 | -1.235 | 0.986 |
|  | UpU - UpH | -0.114 | -0.285 | 0.058 | -2.166 | 0.574 |
|  | UpU – PM | 0.169 | 0.009 | 0.330 | 3.458 | 0.027 |
|  | UpU – MxM | -0.155 | -0.328 | 0.018 | -2.932 | 0.13 |
|  | UpU – UpM | -0.388 | -0.591 | 0.185 | -6.245 | < 0.001 |
|  | PH – MxH | -0.183 | -0.294 | 0.072 | -5.395 | < 0.001 |
|  | PH – UpH | -0.239 | -0.378 | 0.099 | -5.593 | < 0.001 |
|  | PH – PM | 0.044 | -0.070 | 0.159 | 1.262 | 0.984 |
|  | PH - MxM | -0.28 | -0.416 | 0.144 | -6.72 | < 0.001 |
|  | PH – UpM | -0.513 | -0.691 | 0.336 | -9.441 | < 0.001 |
|  | MxH – UpH | -0.056 | -0.202 | 0.091 | -1.241 | 0.986 |
|  | MxH – PM | 0.228 | 0.100 | 0.355 | 5.814 | < 0.001 |
|  | MxH – MxM | -0.097 | -0.240 | 0.046 | -2.215 | 0.539 |
|  | MxH – UpM | -0.33 | -0.512 | 0.148 | -5.919 | < 0.001 |
|  | UpM - PM | 0.283 | 0.130 | 0.436 | 6.03 | < 0.001 |
|  | UpH – MxM | -0.041 | -0.207 | 0.124 | -0.815 | 1 |
|  | UpH – UpM | -0.274 | -0.472 | 0.077 | -4.532 | < 0.001 |
|  | PM – MxM | -0.324 | -0.474 | 0.174 | -7.071 | < 0.001 |
|  | PM – UpM | -0.557 | -0.746 | 0.369 | -9.663 | < 0.001 |
|  | MxM - UpM | -0.233 | -0.430 | 0.036 | -3.862 | 0.006 |
| Elderly | PF – MxF | 0.003 | -0.103 | 0.109 | 0.098 | 1 |
|  | PF – UpF | 0.202 | 0.061 | 0.344 | 4.671 | < 0.001 |
|  | PF – PU | -0.08 | -0.165 | 0.005 | -3.062 | 0.091 |
|  | PF – MxU | -0.039 | -0.136 | 0.058 | -1.299 | 0.979 |
|  | PF – UpU | 0.063 | -0.055 | 0.181 | 1.749 | 0.845 |
|  | PF – PH | -0.211 | -0.288 | 0.133 | -8.905 | < 0.001 |
|  | PF – MxH | -0.17 | -0.260 | 0.081 | -6.203 | < 0.001 |
|  | PF – UpH | -0.095 | -0.209 | 0.019 | -2.736 | 0.209 |
|  | PF – PM | -0.281 | -0.373 | 0.189 | -9.987 | < 0.001 |
|  | PF – MxM | -0.218 | -0.328 | 0.107 | -6.44 | < 0.001 |
|  | PF – UpM | -0.128 | -0.273 | 0.016 | -2.897 | 0.142 |
|  | MxF – UpF | 0.199 | 0.041 | 0.357 | 4.128 | 0.002 |
|  | MxF – UpU | -0.083 | -0.196 | 0.030 | -2.389 | 0.414 |
|  | MxF – MxU | -0.042 | -0.162 | 0.078 | -1.137 | 0.993 |
|  | MxF – UpU | 0.06 | -0.077 | 0.197 | 1.436 | 0.957 |
|  | MxF – PH | -0.214 | -0.322 | 0.105 | -6.433 | < 0.001 |
|  | MxF – MxH | -0.174 | -0.289 | 0.058 | -4.913 | < 0.001 |
|  | MxF – UpH | -0.098 | -0.232 | 0.036 | -2.392 | 0.411 |
|  | MxF – UpM | -0.284 | -0.404 | 0.165 | -7.784 | < 0.001 |
|  | MxF – MxM | -0.221 | -0.353 | 0.089 | -5.459 | < 0.001 |
|  | MxF – UpM | -0.131 | -0.293 | 0.030 | -2.664 | 0.244 |
|  | UpF – PU | -0.282 | -0.428 | 0.136 | -6.315 | < 0.001 |
|  | UpF – MxU | -0.241 | -0.392 | 0.090 | -5.206 | < 0.001 |
|  | UpF – UpU | -0.139 | -0.303 | 0.025 | -2.777 | 0.19 |
|  | UpF – UpH | -0.413 | -0.555 | 0.270 | -9.472 | < 0.001 |
|  | UpF – MxH | -0.373 | -0.520 | 0.225 | -8.27 | < 0.001 |
|  | UpF – UpH | -0.297 | -0.458 | 0.137 | -6.059 | < 0.001 |
|  | UpF – UpM | -0.483 | -0.634 | 0.333 | -10.489 | < 0.001 |
|  | UpF – MxM | -0.42 | -0.580 | 0.260 | -8.581 | < 0.001 |
|  | UpF – UpM | -0.33 | -0.513 | 0.148 | -5.907 | < 0.001 |
|  | PU – MxU | 0.041 | -0.062 | 0.144 | 1.305 | 0.979 |
|  | PU – UpU | 0.143 | 0.020 | 0.266 | 3.794 | 0.008 |
|  | PU – PH | -0.131 | -0.216 | 0.046 | -5.061 | < 0.001 |
|  | PU – MxH | -0.091 | -0.188 | 0.006 | -3.055 | 0.093 |
|  | PU – UpH | -0.015 | -0.134 | 0.103 | -0.425 | 1 |
|  | PU – UpM | -0.202 | -0.299 | 0.104 | -6.738 | < 0.001 |
|  | PU – MxM | -0.138 | -0.254 | 0.022 | -3.891 | 0.006 |
|  | PU – UpM | -0.049 | -0.198 | 0.100 | -1.066 | 0.996 |
|  | MxU – UpU | 0.102 | -0.026 | 0.230 | 2.594 | 0.283 |
|  | MxU – PH | -0.172 | -0.270 | 0.074 | -5.74 | < 0.001 |
|  | MxU – MxH | -0.132 | -0.239 | 0.025 | -4.025 | 0.003 |
|  | MxU – UpH | -0.057 | -0.183 | 0.070 | -1.462 | 0.951 |
|  | MxU – UpM | -0.243 | -0.352 | 0.133 | -7.238 | < 0.001 |
|  | MxU – MxM | -0.179 | -0.304 | 0.055 | -4.718 | < 0.001 |
|  | MxU – UpM | -0.09 | -0.244 | 0.065 | -1.893 | 0.764 |
|  | UpU – PH | -0.274 | -0.393 | 0.154 | -7.493 | < 0.001 |
|  | UpU – MxH | -0.234 | -0.361 | 0.107 | -6.011 | < 0.001 |
|  | UpU - UpH | -0.158 | -0.299 | 0.017 | -3.665 | 0.013 |
|  | UpU – PM | -0.344 | -0.474 | 0.215 | -8.697 | < 0.001 |
|  | UpU – MxM | -0.281 | -0.423 | 0.139 | -6.46 | < 0.001 |
|  | UpU – UpM | -0.191 | -0.359 | 0.024 | -3.729 | 0.01 |
|  | PH – MxH | 0.04 | -0.050 | 0.130 | 1.461 | 0.951 |
|  | PH – UpH | 0.116 | 0.002 | 0.230 | 3.315 | 0.043 |
|  | PH – PM | -0.071 | -0.160 | 0.019 | -2.571 | 0.296 |
|  | PH - MxM | -0.007 | -0.118 | 0.104 | -0.212 | 1 |
|  | PH – UpM | 0.082 | -0.063 | 0.228 | 1.851 | 0.79 |
|  | MxH – UpH | 0.075 | -0.046 | 0.196 | 2.036 | 0.668 |
|  | MxH – PM | -0.111 | -0.213 | 0.008 | -3.537 | 0.021 |
|  | MxH – MxM | -0.047 | -0.165 | 0.070 | -1.319 | 0.977 |
|  | MxH – UpM | 0.042 | -0.108 | 0.192 | 0.917 | 0.999 |
|  | UpM - PM | -0.186 | -0.310 | 0.062 | -4.902 | < 0.001 |
|  | UpH – MxM | -0.123 | -0.259 | 0.013 | -2.948 | 0.125 |
|  | UpH – UpM | -0.033 | -0.196 | 0.130 | -0.666 | 1 |
|  | PM – MxM | 0.063 | -0.057 | 0.184 | 1.715 | 0.862 |
|  | PM – UpM | 0.153 | -0.000 | 0.306 | 3.258 | 0.052 |
|  | MxM - UpM | 0.09 | -0.073 | 0.252 | 1.804 | 0.816 |

Contrasts are based on EMMs from the same beta–logit GLMMs as S1 Table. In the “Contrast” row, P = Paddy-dominated Agricultural area, Mx = Paddy-Upland-Mixed area, Up = Upland-dominated area, F = Flatland area, H = Hilly area, M = Mountainous area, U = Urban area. “Delta (logit)” is the difference on the link (logit) scale; 95% CIs and p-values are multiplicity-adjusted (Tukey). Inference uses asymptotic z-tests (df = ∞). Contrast labels compare two combinations of landtype1 × landtype2 (e.g., “Paddy Flatland − Mixed Flatland”, “PF – MxF”).
