# Supplementary material for: Identifying where Japanese agriculture is most at risk: A longitudinal analytical framework based on municipal boundaries as of 1950 for workforce decline and aging (2005–2020)
Source: PLoS One. 2026 Apr 1;21(4):e0334403. doi: 10.1371/journal.pone.0334403 (PMC13042669; doi:10.1371/journal.pone.0334403)
Supplement: Attachment — Submitted filename: Response to Reviewers.docx [file pone.0334403.s003.docx]

Dear Editor and Reviewers,

We sincerely thank you for your careful and constructive evaluation of our manuscript. We have revised the manuscript accordingly and provide below a point-by-point response to all comments. Revisions include: (i) clarifying the operational definition of hotspot/coldspot and the spatial-lag procedure; (ii) improving methodological transparency by adding detailed MAFF landtype classification rules in the Supporting Information and citing them in the Methods; (iii) revising ambiguous wording and relocating interpretive statements from Results to Discussion where appropriate; and (iv) strengthening the Discussion with targeted citations and a brief addition on food-security implications. All changes are marked in the tracked-changes version, and line numbers in this response refer to the revised manuscript.

**Response to Journal Requirements:**

We thank the Editorial Office for the additional journal requirements and have addressed them as follows.

1. PLOS ONE style requirements (including file naming)

We checked the manuscript formatting and file organization against the PLOS ONE style requirements and templates and revised them as needed. We also confirmed that tables are placed in appropriate locations within the main manuscript text.

2. Financial disclosure / Role of Funder statement

We have prepared an amended Role of Funder statement and included it in the cover letter, as requested.

3. Copyright and licensing of map figures (Figs. 1, 3, 4, 5, 7, and 8)

We addressed the map copyright/licensing requirement by confirming the terms of use of all geospatial data sources used in this manuscript. All map figures (Figs 1, 3, 4, 5, 7, and 8) were created by the authors in QGIS using publicly available Japanese government geospatial datasets, including MAFF administrative boundary data and the MLIT National Land Numerical Information data. The MAFF data were used in accordance with the MAFF Terms of Use (https://www.maff.go.jp/e/use/term_use.html), which state that MAFF website content is subject to the Public Data License (PDL) 1.0 (<https://www.digital.go.jp/>

en/resources/open_data/public_data_license_v1.0). PDL 1.0 explicitly states compatibility with CC BY 4.0. The MLIT National Land Numerical Information download service terms of use also explicitly state compatibility with CC BY 4.0 (https://nlftp.mlit.go.jp/ksj/other/agreement.html). Accordingly, we believe that the map figures in our manuscript are compatible with PLOS ONE’s CC BY 4.0 publication requirements. We have revised the manuscript to clarify the data sources and the licensing basis including the relevant terms-of-use pages in the Methods section and source descriptions.

4. Tables in the main manuscript

The tables were already included in the main manuscript. We re-checked their placement and confirmed that they are inserted at appropriate locations in the text. Supplementary tables remain as Supporting Information files, as appropriate.

5. Supporting Information captions and in-text citations

We added the Supporting Information file captions at the end of the main manuscript and updated the corresponding in-text citations accordingly.

7. Reviewer-suggested references

In line with the reviewer comments, we evaluated additional relevant published studies and added citations where appropriate. Please see our detailed responses (especially Reviewer #2, Q8) for the specific revisions.

8. Reference list check

We reviewed the reference list for completeness and accuracy and corrected/updated entries where needed. Any relevant changes are described in the response letter.

**Response to Reviewer #1**

**Q1**

**Line 57: Why 2005 and 2020 are selected should be described.**
A1

Thank you for the comment. We agree that the rationale should be stated explicitly. To avoid interrupting the general problem framing in the opening of the Introduction, we clarified this point in the Methods (Data source) where the study period is defined. Specifically, we note that a major revision in data collection was implemented in 2005, which changed the statistical definitions/coverage, and therefore continuous and comparable census datasets are available from 2005 onward (Lines 125-127).

**Q2**

**Line 108: Need to define ‘hotspot’.**

A2

Thank you for this comment. We agree that the term “hotspot” can be ambiguous without an explicit operational definition. In the revised manuscript, we therefore rephrased the Introduction to avoid using “hotspot” in the study objective and instead refer to “spatial clusters where decline and aging are most pronounced,” and defined “hotspot” and “coldspot” explicitly in the Methods. Specifically, we compute distance-weighted spatial-lag scores within a 50-km neighborhood using a linear taper (triangular) kernel (excluding the focal unit), standardize these scores to spatial-lag Z-scores, and then transform them into a rank-based 0–1 scale using empirical ranks. We operationally define hotspots as the top decile and coldspots as the bottom decile of this national rank distribution for each indicator. (Lines 109-110 and 245-257)

**Q3**

**Lines 221-222: Explain how these topographical categories were determined.**

**Lines 222-223: Explain how these land-use types were determined. “as defined by the MAFF” is not enough**

**Line 232: Explain landtype1 and landtype 2.**

A3

Thank you for this suggestion. In the revised manuscript, we clarify that landtype1 (topographical categories) and landtype2 (land-use types) follow the MAFF Agricultural Area Classification and form a nested structure. Because the full determination procedure depends on MAFF’s published indicator definitions, decision order, and threshold rules, we added a new Supporting Information section (S1 Appendix) that documents these criteria in detail, and we inserted an explicit in-text reference to S2 Appendix at the point where landtype1 and landtype2 are introduced in the Methods (Lines 229-231).

**Q4**

**Line 260: ‘workforce size’ is not clear.**

A4

Thank you for the comment. We agree that “workforce size” was potentially ambiguous. We have revised the sentence to use the more explicit wording “the number of workers” (Lines 278-279).

**Q5**

**Lines 312-316: These are not result and should be transferred to Discussion.**

A5

Thank you for this suggestion. We agree that the original wording included interpretive statements that are more appropriate for the Discussion. In the revised manuscript, we rewrote this passage in the Results to report only the observed spatial patterns shown in Figs. 4-5 and removed interpretive phrasing regarding sustainability and broader structural implications, which are discussed in the Discussion section (Lines 334-339).

**Q6**

**Line 351: What is ×?**

A6

Thank you for pointing this out. In the revised manuscript, we clarified that the symbol was used only to indicate the combination of topography (landtype1) and land-use type (landtype2). To avoid any confusion with multiplication or statistical notation, we replaced “×” with an en dash (e.g., “Flatland–Paddy”) and consistently refer to these as “combinations” throughout the Results (Lines 376-384).

**Q7**

**Line 395: “broadly across Tohoku” is confusable, because you mention that the northernmost and southeastern parts of Tohoku are hotspots.**

A7

Thank you for this helpful comment. We agree that the original wording could be confusing because “Tohoku” appears in descriptions of both indicators. To prevent any misinterpretation, we revised the text to explicitly label coldspots by outcome. In the revised manuscript, we now refer to “Declining coldspots” for the decline-rate map (Fig. 7) and “Elderly coldspots” for the aging map (Fig. 8), clarifying that the hotspot locations in northernmost and southeastern Tohoku pertain to the decline rate, whereas the statement “broadly across Tohoku” refers to coldspots in the proportion of elderly workers(Lines 419 and 422).

**Response to Reviewer #2:**

**1. I have really enjoyed reviewing this manuscript, especially the methodology section. The methodology has a very solid foundation. The detailed explanation of stages taken to arrive at sub-municipalities makes it easy for other researchers to replacate the study in both Japan and beyond.
2. The problem statement is well grounded and persuasively and convincingly shows clear research gap that was being addressed by the study.
3. Even though I have not had access to some of the figures, it is clear from the presentation of the statistics that the statistical analysis was both rigorous and robust.
4. The results are easy to follow even to non-scientists which is good for wider readership.**

Thank you very much for your positive and encouraging comments on the manuscript. We are pleased that you found the methodological framework solid and clearly described, and that the problem statement effectively articulated the research gap. We also appreciate your assessment of the rigor of the statistical analysis and the clarity of the results presentation for a broad readership

**Q8**

**5. The discussion of the results should be improved in terms of relating the results to previous studies but also linking them to relevant theories. In fact, to me this is the minor but important issue that the authors should consider addressing. In other words, while the study is making very important methodological contributions, it has remained silent on its theoretical underpppings and theoretical contributions.**

A8

Thank you for this constructive suggestion. In the revised manuscript, we strengthened the Discussion by more explicitly relating our empirical findings to prior studies addressing mechanisms and contextual factors underlying agricultural workforce decline and aging. Specifically, we added targeted citations to support interpretations regarding (i) the co-occurrence of workforce decline with geographic constraints and labor-intensive farming conditions in mountainous and upland-dominated areas [48,49,54], (ii) peri-urban patterns where proximity to consumer markets may contribute to the retention of agricultural workers [50], (iii) the role of cross-sector labor-market conditions in shaping agricultural viability [51], (iv) the shocks by the 2011 Fukushima nuclear disaster to localized workforce decline [52], and (v) the linkage between aging, constraints on generational replacement, and structural conditions limiting entry and continued farming in disadvantaged regions [53]. We also expanded the policy discussion with references concerning pathways for sustaining output through investment and mechanization [55], the potential vulnerability associated with spatial concentration of domestic supply under region-specific shocks [56], and the dependence of rural settlement functions on agricultural labor and collective maintenance [57,58].

Our primary aim in this paper is to establish a nationwide analytical framework and empirical baseline at the sub-municipality scale. Accordingly, rather than advancing or testing a specific theoretical model, we positioned our findings within the existing empirical and conceptual literature through these additional citations, thereby clarifying how the observed spatial patterns align with and inform prior work.

**Q9**

**6. Both conclusions and policy implications are speaking and well connected to results of the study. This is commendable given that it is not uncommon to see research conclusions and policy recommendations that are completely disconnected from research findings. However, it will do the paper more justice if a discussion is made on implications of the results on as far as food security in Japan in concerned.**

A9

Thank you for this positive assessment and for the helpful suggestion. In the revised Discussion, we added text linking our spatial findings to food-security considerations by noting that policies aimed at sustaining output in selected areas (e.g., through capital investment and mechanization) may also intensify spatial concentration of domestic supply and thereby increase vulnerability to region-specific shocks (e.g., extreme weather and earthquakes). We also added a brief statement highlighting that the high prevalence of workers aged ≥75 in paddy-dominated areas has direct implications for staple rice production, because workforce contraction as this cohort exits farming could challenge longer-term continuity of domestic rice supply without targeted measures to support succession and structural adaptation (Lines 509-511 and 533-535).

**7. I have attached the manuscript with my comments in track changes.**

**Q10**

**Lines 81-92 (MC1) The problem statement is well grounded the research gap excellently exposed**

A10

Thank you for this positive assessment.

**Q11**

**L205-207 (MC2) It is important also to discuss this in the context retirement age for a typical Japanese employed in non-agricultural occupations. For example, after the off 65, what proportion continues to work in non-agricultural jobs after attaining the 75 years age? This will provide an opportunity for a nuanced comparative analysis of proportion of the elderly in core agricultural workers versus their proportion among non-core agricultural workers (i.e. those employed elsewhere)**

A11

Thank you for this suggestion. We agree that interpreting the ≥75-year threshold benefits from placing it in the broader context of work at advanced ages in Japan. In the revised manuscript, we clarified that our “elderly” indicator is a composition-of-workers metric (i.e., the share of individuals aged ≥75 within a given workforce). We then added an economy-wide benchmark using the same metric: in 2020, workers aged ≥75 accounted for about 3.52% of all employed persons in Japan (2020 Population Census) [37]. We incorporated this benchmark in the Methods and used it in the Discussion to highlight that employment at ages ≥75 is uncommon in the overall workforce, thereby supporting our use of ≥75 years as a conservative indicator of advanced aging among core agricultural workers (Lines 211-214, 476-478).

**Q12**

**L243 (MC3) What were the cut off points (thresholds) for categorizing areas as hotspots or coldspots**

A12

Thank you for this comment. We defined hotspots and coldspots using percentile thresholds of the local neighborhood Z-scores after conversion to empirical percentiles. Specifically, hotspots correspond to the upper 10% (≥90th percentile) and coldspots to the lower 10% (≤10th percentile) of the national distribution (computed separately for each indicator and year). We clarified this in the Methods (Lines 245-255).

**Q13**

**L312 (MC4) This adverb is hanging here**

A13

Thank you for pointing this out. In the revised manuscript, we rewrote the sentence for clarity by integrating the modifier into a complete phrase eliminating the hanging adverb and improving readability (Line 326, see also Reviewer #1, Q5).

**Q14**

**Lines 378-379 (MC5) Perhaps this addresses my earlier question on thresholds**

A14

Thank you for this note. Yes, this point is addressed in the revised manuscript. In response to Reviewer #1 (Q2), we revised and clarified the hotspot/coldspot definition in the Methods section. We now explicitly state that hotspots and coldspots are defined using percentile thresholds of the empirical national distribution of the local neighborhood Z-scores (computed separately for each indicator and year), with hotspots as the top decile (≥90th percentile) and coldspots as the bottom decile (≤10th percentile).

**Q15**

**Line 458 (MC6) Why limited entry for young workers? Is because young people do not find farming to be cool and fashionable or because the initial capital to enter farming is unaffordable to them? By the way, how does Japan census/stats categorize young people who are working full time in their elderly parents’ farms? Are they also treated as core agricultural workers?**

A15

Thank you for these questions. We agree that the phrasing “limited entry of younger workers” required clearer contextualization and should not be interpreted primarily as an attitudinal explanation (e.g., whether farming is perceived as “cool” or “fashionable”). In Japan, the underlying population structure itself has become strongly top-heavy, with substantially fewer

young cohorts relative to older cohorts, which constrains new labor-market entry across industries, not only in agriculture. In the revised Discussion, we therefore reframed this point by explicitly positioning reduced entry of younger workers as a broad demographic backdrop in Japan, whose magnitude varies across regions, and by emphasizing that spatial differences largely hinge on whether farming can be sustained by older workers and whether local conditions facilitate younger entry/retention. We also clarified the contrast between paddy-dominated flatlands, where mechanization and infrastructure can allow older farmers to continue production despite limited generational replacement, and mountainous areas, where geographic and market constraints are likely to restrict both succession and continued farming, resulting in co-occurring aging and workforce decline (Lines 487-503).

Regarding classification, the agricultural census defines core agricultural workers based on an individual’s usual status and principal occupation. Accordingly, as long as the farm is an individual (family-run) management entity rather than an organizational entity (an agricultural corporation or cooperative), younger family members who work full-time on their parents’ farms are counted as core agricultural workers. This definition and the scope of the target population (individual entities vs. organizational entities) are described in the manuscript (Lines 129–146).
